# A Metabolomics-Inspired Strategy for the Identification of Protein Covalent Modifications

**DOI:** 10.3389/fchem.2019.00532

**Published:** 2019-07-31

**Authors:** João Nunes, Catarina Charneira, Carolina Nunes, Sofia Gouveia-Fernandes, Jacinta Serpa, Judit Morello, Alexandra M. M. Antunes

**Affiliations:** ^1^Centro de Química Estrutural, Instituto Superior Técnico, Universidade de Lisboa, Lisbon, Portugal; ^2^CEDOC, Chronic Diseases Research Centre, Faculdade de Ciências Médicas, NOVA Medical School, Universidade NOVA de Lisboa, Lisbon, Portugal; ^3^Unidade de Investigação em Patobiologia Molecular do Instituto Português de Oncologia de Lisboa Francisco Gentil, Lisbon, Portugal

**Keywords:** adductomics, metabolomics, mass spectrometry, chemometrics, toxicology, acrylamide, glycidamide, histones

## Abstract

Identification of protein covalent modifications (adducts) is a challenging task mainly due to the lack of data processing approaches for adductomics studies. Despite the huge technological advances in mass spectrometry (MS) instrumentation and bioinformatics tools for proteomics studies, these methodologies have very limited success on the identification of low abundant protein adducts. Herein we report a novel strategy inspired on the metabolomics workflows for the identification of covalently-modified peptides that consists on LC-MS data preprocessing followed by statistical analysis. The usefulness of this strategy was evaluated using experimental LC-MS data of histones isolated from HepG2 and THLE2 cells exposed to the chemical carcinogen glycidamide. LC-MS data was preprocessed using the open-source software MZmine and potential adducts were selected based on the *m/z* increments corresponding to glycidamide incorporation. Then, statistical analysis was applied to reveal the potential adducts as those ions are differently present in cells exposed and not exposed to glycidamide. The results were compared with the ones obtained upon the standard proteomics methodology, which relies on producing comprehensive MS/MS data by data dependent acquisition and analysis with proteomics data search engines. Our novel strategy was able to differentiate HepG2 and THLE2 and to identify adducts that were not detected by the standard methodology of adductomics. Thus, this metabolomics driven approach in adductomics will not only open new opportunities for the identification of protein epigenetic modifications, but also adducts formed by endogenous and exogenous exposure to chemical agents.

## Introduction

Protein covalent adducts, which can either result from exposure to endogenous or exogenous chemical electrophiles (i.e., non-enzymatic) or be enzymatically driven (i.e., post translational modifications- PTMs), have a key role at the onset of multiple health issues, including cancer and immune effects (Nunes et al., 2016; Gonzalez-Morena et al., 2017). Therefore, adductomics studies focused on the identification of key adducted proteins, on the nature and the extent of the covalent modification, along with the identification of the sites of adduction within the protein, represent a huge opportunity for a better understanding of events underlying diseases and chemically-induced adverse reactions.

As part of our research program on understanding the role of histones covalently modified by chemical carcinogens in the onset of chemically-induced cancer, we were challenged to overcome the major analytical limitation of adductomics studies: the fraction of adducted proteins is very low when compared with non-adducted proteins *in vivo*. Namely, human serum albumin (HSA) adducts occur at 0.1 mol% levels, or less, *in vivo* (reviewed by Sabbioni and Turesky, 2017). A frequent strategy to overcome this issue is to monitor the formation of covalent adducts in targeted (hot-spots) residues of proteins, using multiple reaction monitoring (MRM) acquisition to target specific parent and fragments ions (reviewed by Nunes et al., 2019). Despite the indisputable role of such approaches for the identification of biomarkers of exposure (reviewed by Carlsson et al., 2019), they are ineffective in providing information on the underlying mechanisms of the chemically-induced adverse reactions. These specific studies demand not only the identification of adducted proteins that have toxicological roles but also which residues on the protein were modified. The major trend in such investigations is to adopt the MS-based shotgun proteomics workflows that traditionally rely on the chromatographic separation of digested peptides followed by a data dependent analysis (DDA), where MS and MS/MS data of selected precursors are afforded in a single run, thereby allowing subsequent adduct identification using database search engines that compare experimental and theoretical MS/MS spectra (reviewed by Gan et al., 2016; Tailor et al., 2016; Sabbioni and Turesky, 2017). Despite this workflow has been successfully applied to adductomics studies for the identification of high-abundant covalent adducts (reviewed by Nunes et al., 2019), it is easy to understand the failure of this strategy in the identification of low-abundant adducted peptides *in vivo* and *ex vivo*. In fact, by DDA methods only 10% of detectable peptides are identified and these methods are linked with low reproducibility across runs (Michalski et al., 2011). Basically, by this methodology we must be extremely fortunate for the parent ion of a covalently-modified peptide to be picked for MS/MS analysis, in at least one sample, enabling its subsequent identification by proteomics search engines. Data Independent Analysis (DIA) strategies (reviewed by Law and Lim, 2013) emerged to overcome DDA reproducibility and sensitivity drawbacks by fragmenting all peptides in a given *m/z* window. However, while this approach presents major advantages for proteomics studies (reviewed by Vidova and Spacil, 2017), its applicability to adductomics studies is still limited and focused mostly in targeted-peptide site-specific modifications (Bruderer et al., 2015; Porter and Bereman, 2015; Carlsson et al., 2017).

The failure of such MS-based strategies on the identification of toxicologically relevant low abundant adducted protein residues is not a result of instrumental limitations but rather of the lack of adequate analytical workflows for these specific adductomics studies. Actually, while dealing with proteins, the goals of adductomics are completely distinct from the ones of proteomics studies. Adductomics, is not aimed at observing alterations on the protein profiles. Instead, it is focused on identifying adducted peptides (which can be considered small molecules) that are present (or are more abundant) in a given population of samples but absent (or less abundant) in control samples. This is much closer to the metabolomics goals than to the proteomics ones. Therefore, this led us to propose a novel approach inspired by the workflow commonly used in metabolomics studies consisting on LC-MS data preprocessing followed by statistical analysis (reviewed by Katajamaa and Oresic, 2007; Dunn et al., 2011).

**Figure 1:**
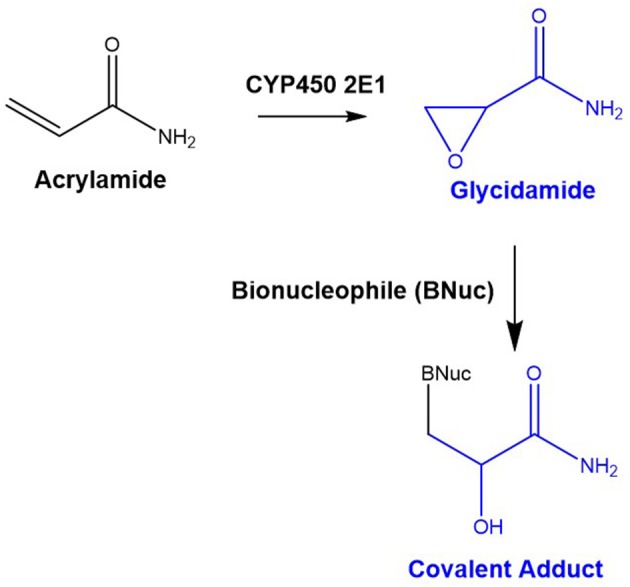
Acrylamide bioactivation to glycidamide and formation of stable covalent adducts with bionucleophiles. Glycidamide stems from cytochrome P450 isoform 2E1 (CYP 2E1)-catalyzed oxidation of acrylamide, and bionucleophiles (DNA bases and amino acid residues bearing a nucleophilic side chain) can promote the ring-opening reaction of this reactive metabolite yielding stable covalent adducts.

To test the applicability of this strategy, two distinct types of hepatic cell lines, the tumorigenic HepG2 (ATCC® HB-8065™) and the non-tumorigenic THLE-2 (ATCC® CRL2706™), were exposed to distinct doses of glycidamide. This epoxide is the reactive metabolite responsible for the carinogenic effects of the environmental and food pollutant acrylamide (Beland et al., 2015) and is known to react with proteins and DNA yielding covalent adducts that are stable under enzymatic, chemical and thermal digestion/detachment pre-analysis conditions (Wilson et al., 2009; Von Tungeln et al., 2012; Beland et al., 2015) (Figure 1), thereby enabling their subsequent identification by MS-based methodologies. Histones isolated from these cell lines were digested to peptides and analyzed by LC-MS in DDA mode, which produced both MS and MS/MS data. The open source software MZmine (Katajamaa et al., 2006; Pluskal et al., 2010) was used for LC-MS data preprocessing and only those ions with *m/z* increments corresponding to glycidamide incorporation were selected for statistical analysis. Multivariate analysis was then performed to select those ions differently present in cells exposed and not exposed to glycidamide. The results of our method were compared with the ones obtained upon analysis of MS/MS data obtained from DDA with the proteomic data search engines Mascot, Global Proteome Machine interface (GPM Fury) (Beavis, 2006) that uses X!Tandem (Craig and Beavis, 2004), MaxQuant (Craig and Beavis, 2004), and MSFragger (Kong et al., 2017).

## Materials and Methods

### Cell Cultures

Two liver cell lines were used in this study: the non-tumorigenic cell line THLE-2 (ATCC® CRL2706™) and the hepatocellular carcinoma cell line HepG2 (ATCC® HB-8065™). Both cell lines were obtained from the American Type Culture Collection (ATCC).

Cells were maintained at 37°C in a humidified 5% CO_2_ atmosphere. THLE2 cells were cultured in BEGM (CC-3170; Lonza™) plus the provided supplements and following the indications by ATCC, and supplemented, in addition, with 10 % FBS (S 0615; Invitrogen™, Life Technologies) and 1% antibiotic-antimycotic (15240062; Invitrogen™, Life Technologies). HepG2 cells were cultured in DMEM 1X (41965-039; Invitrogen™, Life Technologies) supplemented with 10% FBS and 1% antibiotic-antimycotic. Prior to any experiment, cells were synchronized under starvation (FBS free culture medium) overnight.

### Glycidamide Exposure and Sampling

Glycidamide (Sigma-Aldrich) was prepared at 1M in DMSO. Cells were incubated with glycidamide at 0.1, 1, and 5 mM for 16 h. For each experiment, two control conditions were applied: control medium or DMSO 0.1% (v/v). For both cell lines, 2 × 10^6^ cells cultured in a 125 cm^2^ T-flask were used in each replicate of all culture conditions. The number of replicates for each cell line and condition was between 3 and 6.

### Histone Isolation and Digestion

Nuclear histones were isolated by an adaptation of the methodology described by Lin and Garcia (2012). Specifically, cells were first resuspended in nuclei isolation buffer (15 mM Tris–HCl pH 7.5, 60 mM KCl, 15 mM NaCl, 5 mM MgCl_2_, 1 mM CaCl_2_, 250 mM sucrose, and 0.2% NP-40) supplemented with the following inhibitors: 1 mM DTT, 0.5 mM AEBSF, and 10 mM sodium butyrate. The suspension was subsequently centrifuged (1,000 × g for 5 min at 4°C) and supernatants were discarded. The nuclear fraction was washed with nuclei isolation buffer (without NP-40) and centrifuged (1,000 × g for 5 min at 4°C). Pellets were subsequently homogenized in 0.4 N H_2_SO_4_ and incubated at 4°C with shaking for 2 h. The nuclei were pelleted at 3,400 × g for 5 min, and proteins were precipitated from the supernatant with 25% TCA (w/v) for 1 h at 4°C. The pellet was then washed with pure acetone to remove residual TCA. Protein concentrations were assessed by the Bradford assay.

Histones were digested with trypsin in a 50 mM ammonium bicarbonate buffer for 2 h (with a 1:10 w/w trypsin/histone ratio) (Nunes et al., 2016). The digestions were quenched by addition of formic acid.

### Liquid Chromatography-High Resolution Mass Spectrometry (LC-HRMS)

Following histones digestion, the peptides were analyzed by liquid chromatography (Ultimate 3000 RSLCnano system, Thermo Scientific, Bremen, Germany) interfaced with a Bruker Impact II quadrupole time-of-flight mass spectrometer equipped with a CaptiveSpray (nanospray) source (Bruker Daltoniks, Bremen, Germany). Chromatographic separation was performed on an Acclaim PepMap C18 column (75 μm × 150 mm, 3 μm particle size; Thermo Scientific). The mobile phase consisted of water containing 0.1% formic acid (A) and acetonitrile:water (80:20) containing 0.1% formic acid (B). The elution conditions were as follows: 2% B for 5 min, 2–50% B over 45 min, 50–60% B over 10 min, 60–65% B over 5 min, 95–2% B over 3 min, and 2% B for 27 min. The injection volume was 1 μL, the flow rate was 300 nL/min, and the column was maintained at 40°C. Quality control samples (a tryptic peptide digest of bovine serum albumin) were analyzed along with the analytical runs (after every 10 samples) in order to check the consistency of analysis regarding signal intensity and retention time deviations. A Lock Mass (HP-121 Calibration Standard, *m*/*z* 1221.9906; Agilent Technologies, Santa Clara, CA, U.S.A.) was used during the analysis for spectrum calibration. Data were acquired in positive mode from *m/z* 100 to 2200 at an acquisition rate of 5 spectra/sec, using a data-dependent auto-MS/MS method to select the 10 most abundant precursor ions per cycle for fragmentation. The MS source parameters were set as follows: dry gas heater temperature, 150°C; dry gas flow, 3 L/min; and capillary voltage, 1600 V.

### LC-MS Data Preprocessing Followed by Statistical Analysis

#### LC-MS Data Preprocessing

The acquired LC-MS data files were converted to ^*^.mzXML files using the ProteWizard MSconvert tool (Chambers et al., 2012). LC-MS data was then preprocessed with the open-source software MZmine (Katajamaa et al., 2006; Pluskal et al., 2010) and consisted of peak detection, removal of isotopes, correction of retention time, peak matching and peak filling.

Peak detection was performed in three steps: (i) mass detection with noise value = 20,000 and retention time range = 17–48 min; (ii) chromatogram builder with minimum time span = 0.2 min, minimum height = 20,000 and *m/z* tolerance = 0.005 Da or 15 ppm; (iii) deconvolution with peak width = 0.2–1.5 min, noise = 20,000. Isotopes were removed using the isotopic peak grouper with *m/z* tolerance = 0.005 Da or 10 ppm, retention time tolerance = 3.5 min and minimum standard intensity = 20,000. Then, a filter was applied to keep only those ions with at least 2 peaks in their isotope pattern. Retention time was corrected with *m/z* tolerance = 0.005 Da or 10 ppm, retention time tolerance = 3.5 min and minimum standard intensity = 20,000. Peak matching among samples was performed using the RANSAC aligner with *m/z* tolerance = 0.005 Da or 10 ppm, retention time tolerance before and after correction = 3.5 and 2 min respectively, RANSAC iterations = 0, minimum number of points = 40%, threshold value = 3.5 and required same charge state. Gap filling was applied using the method peak finder with retention time correction with intensity tolerance = 40%, *m/z* tolerance = 0.005 Da or 10 ppm, retention time tolerance = 1 min.

Taking into consideration that under ESI ionization multicharged ions are obtained for peptides, only ions with charge larger or equal to +2 were kept. The resulting list was split by ion charge (+2, +3, and +4). Potential adducts were searched for each ion charge list using the adduct search tool of MZmine. Knowing the mass increment of 87.0320 between the non-modified peptide and the glycidamide-modified peptide, adduct search was performed for increments in *m/z* of 43.516, 29.011 and 21.758 for ions with charge +2, +3 +4, respectively (*m/z* tolerance = 0.005 Da or 10 ppm, maximum relative adduct peak height = 70%). A final filter was applied to keep only those potential adducts identified in at least half of the samples exposed to the highest dose of glycidamide in each cell line.

#### Statistical Analysis

Data was centered and unit variance scaled before statistical analysis. Multivariate analysis was performed using Principal Component Analysis (PCA) and Partial Least Square (PLS) Analysis with SIMCA software package version 14.1 (MKS Umetrics, Umeå, Sweden).

### MS/MS Data Processing by Proteomics Search Engines

The acquired MS data files of the samples exposed to the highest dose of glycidamide were converted to ^*^.mgf format using the Compass DataAnalysis software (Bruker Daltonics). Four distinct search engines were used for peptide identification: Mascot (v2.6, Matrix Science Ltd., London, UK) (Perkins et al., 1999), the Global Proteome Machine interface (GPM Fury) (Beavis, 2006) that uses X!tandem (Craig and Beavis, 2004), MaxQuant (Cox and Mann, 2008) and MSFragger (Kong et al., 2017). Search parameters were the same for all four methods and included precursor ion mass tolerance = 15 ppm, fragment ion mass tolerance = 30–40 ppm, number of missed-cleavages ≤3 and variable amino acid modifications = oxidation of methionine and glycidamide incorporation (mass increment of 87.0320 Da) at the most nucleophilic amino acids, namely lysines, cysteines, serines, histidines, and arginines. The acquired MS/MS spectra was searched against an *in-house* compiled human histones database. All human histones sequences were obtained from Uniprot (UniProt Consortium, 2007). Lastly, only in the case of MSFragger, the generated pepXML files were processed by Peptide Prophet (Keller et al., 2002) via the Trans-Proteomic Pipeline (v5.1.0) (Deutsch et al., 2010) with the following settings: use accurate mass binding using PPM, use a non-parametric model and report decoy hits.

Several filters were applied to each search engine. For Mascot and X!Tandem, the significance threshold was set to *p* < 0.05. Additionally, in Mascot, only peptides identified with Mascot Ion Score > 13 were considered. For MaxQuant, the minimum score for modified peptides was set to 40. For MSFragger, the list of peptides obtained after running the Peptide Prophet, was filtered with a peptide probability>0.9. Nonetheless, only those peptides containing glycidamide in at least half of the samples exposed to the highest dose of glycidamide in each cell line were considered. All spectra corresponding to glycidamide-modified peptides were manually checked.

### Stoichiometric Ratios

After adducts identification, stoichiometric ratios between the peak area of each adduct and corresponding non-modified peptide were calculated using MZmine. Targeted peak detection was performed with the list of the adducted and non-modified peptides containing their *m/z* and retention times and the following parameters: shape tolerance = 10%, noise level = 1000, *m/z* tolerance = 0.005 or 15 ppm, retention time tolerance = 3.5 min. Peak matching among samples was performed using the RANSAC aligner with *m/z* tolerance = 0.005 Da or 10 ppm, retention time tolerance before and after correction = 3.5 and 2 min, respectively, RANSAC iterations = 0, minimum number of points = 10%, threshold value = 3.5. Gap filling was applied using the method peak finder with retention time correction with intensity tolerance = 10%, *m/z* tolerance = 0.005 Da or 10 ppm, retention time tolerance = 1 min.

## Results and Discussion

### Metabolomics-Inspired Approach: LC-MS Data Preprocessing Followed by Statistical Analysis

LC-MS data was preprocessed in five steps: peak detection, removal of isotopes, correction of retention time, peak matching and peak filling. This initial processing step generated a list of 20,250 ions, with their corresponding *m/z*, ion charge, retention time and peak area for each sample. Potential adducts were subsequently extracted by the adduct search tool of MZmine, using the mass increment of 87.0320 corresponding to glycidamide incorporation. This procedure led to a list of 718 ions corresponding to potential adducts. Modified-peptides resulting from glycidamide incorporation are expected to be observed in at least half of the samples exposed to the highest dose of glycidamide in each cell line. This enabled to reduce the number of ions corresponding to potential glycidamide-modified peptides to a final list of 57 ions.

**Figure 2:**
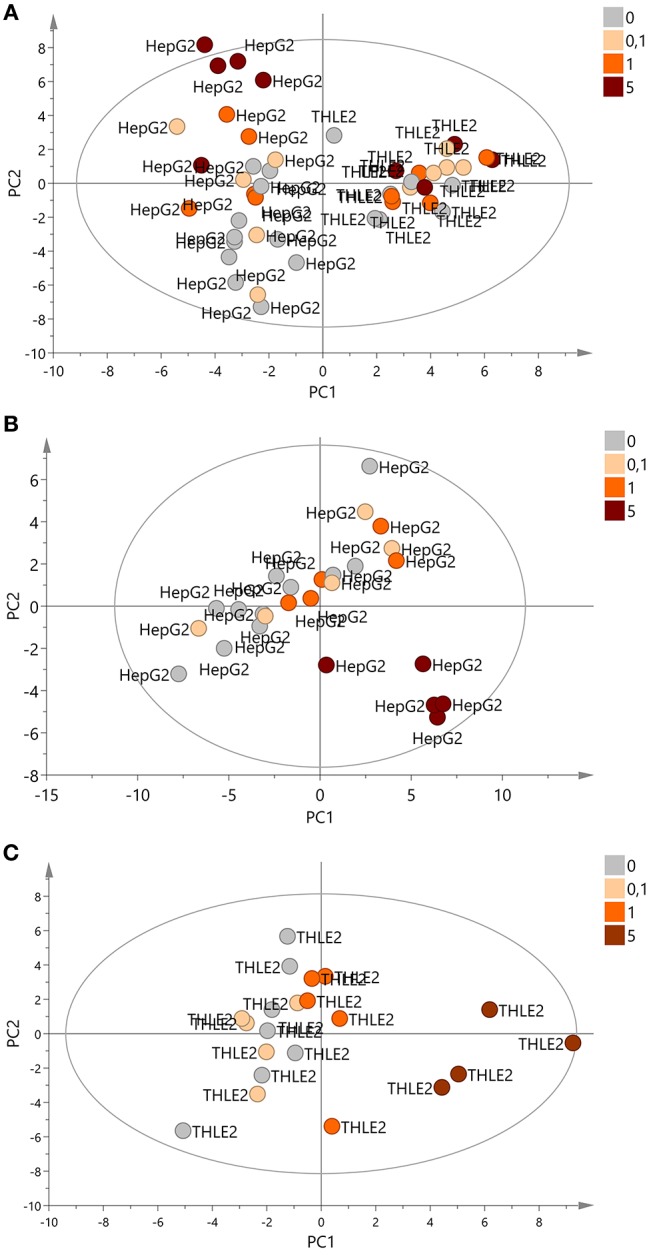
Score plots of PCA. **(A)** PCA with all samples (*n* = 47). First two components covered 22 and 19% of the variance of the data, respectively. **(B)** PCA with HepG2 cells (*n* = 26). First two components covered 31 and 14% of the variance of the data, respectively. **(C)** PCA with THLE2 cells (*n* = 21). First two components covered 21 and 16% of the variance of the data, respectively. Samples were colored according to glycidamide concentration and labeled according to the cell line.

After data preprocessing, multivariate analysis was performed with the 47 samples and 57 potential adducts. Principal Component Analysis (PCA) was used to identify the principal sources of the variance of the data. Cell type had a clear influence in the data, while glycidamide exposure seemed to have little influence (Figure 2A). Then, PCA models were built for each cell line. Samples exposed to the highest concentration of glycidamide were clustered apart from the rest of the samples in both HepG2 and THLE-2 cell lines (Figures 2B,C). Additionally, THLE-2 cells exposed to 1 mM appeared also separated from controls and cells exposed to 0.1 mM of glycidamide (Figure 2C).

In view of these results, partial least square (PLS) analysis was conducted for each cell line with glycidamide concentration as the dependent variable. The PLS model for HepG2 cells had 2 components that explained 45% of the X variation and 93% of the Y variation (Supplementary Figure 1A). The PLS model for THLE-2 cells had 1 component that explained 21% of the X variation and 93% of the Y variation (Supplementary Figure 1B). Both models were statistically significant (*p* < 0.001). In order to identify the peptides affected by glycidamide concentrations (glycidamide adducts), variable importance on the projection (VIP) values were obtained for each ion. Those ions with a VIP value > 1.5 (value chosen empirically) were selected as potential glycidamide adducts: *n* = 9 for HepG2 cells and 10 for THLE-2 cells. Extracted ion chromatograms were obtained for each potential adduct and its corresponding non-modified peptide to check their chromatographic shape and isotopic distribution in controls and cells exposed to the highest concentration of glycidamide. Five ions from the lists of each cell line were discarded due to noise like chromatogram, identification of the ion in control samples or non-correct ion charge assignment. The final list of ions was manually checked to confirm their absence from control samples, consisting of the same cell lines only exposed to cell medium or to cell medium and DMSO (the solvent used to dilute glycidamide), thereby supporting the fact that these ions corresponded to glycidamide-adducted peptides. The final list of glycidamide adducts contained 4 common ions for both HepG2 cells and THLE-2 with *m/z* 565.7740, 580.3200, 627.6670, and 754.0300 and 1 unique ion for THLE-2 cells with *m/z* 467.0030 (Table 1).

**Table 1:** Comparison of results obtained by our novel metabolomics-inspired and the standard Adductomics strategies.

**Cell Line**	**Glycidamide-modified peptide**	**Mass (daltons)**	***m/z* ± error (ppm) (charge)**	**Protein**	**Novel approach**	**GPM Fury**	**MaxQuant**	**MASCOT**	**MSFragger**
HepG2	^110^**H**AVSEGTKAVTKYTSSK^126^	1879.9639	627.6670 ± 8.12 (+3)	Histone H2B			——		
	^74^IAGEASRLAHYNKRSTITSR^93^	2317.2251	580.3200 ± 11.03 (+4)	Histone H2B					
	^2^TKIKADPDGPEAQAEA**C**SGER^22^	2259.0437	754.0300 ± 10.74 (+3) 565.7740 ± 10.25 (+4)	H/ACA ribonucleoprotein complex subunit 2					
THLE-2	^110^**H**AVSEGTKAVTKYTSSK^126^	1879.9639	627.6670 ± 8.12 (+3)	Histone H2B					
	^110^**H**AVSEGTKAVTKYTSAK^126^	1863.9690	467.0030 ± 7.49 (+4)	Histone H2B					
	^74^IAGEASRLAHYNKRSTITSR^93^	2317.2251	580.3200 ± 11.03 (+4)	Histone H2B					
	^2^TKIKADPDGPEAQAEA**C**SGER^22^	2259.0437	754.0300 ± 10.74 (+3) 565.7740 ± 10.25 (+4)	H/ACA ribonucleoprotein complex subunit 2					

*Modified peptides identified by each methodology in at least half the samples exposed to the highest dose of glycidamide are highlighted in green. Cells marked in red represent peptides that were not identified, in at least half the samples exposed to the highest dose of glycidamide, by each methodology. Bold and underline aminoacids represent the glycidamide binding site*.

*Uniprot access numbers of each histone variant corresponding to the identified peptides are given in Supplementary Table 1*.

### Comparison Between Our Novel Metabolomics-Inspired and the Standard Adductomics Approaches

MS/MS data obtained from DDA of samples exposed to the highest dose of glycidamide were analyzed by four distinct database-dependent methods (Mascot, GPM Fury, MaxQuant and MSFragger) for the identification of glycidamide-modified peptides. The proteomics data search engines GPM Fury and MaxQuant enabled the identification of three glycidamide-modified peptides in at least half of the samples exposed to the highest dose of glycidamide in each cell line. Mascot only identified two glycidamide-modified peptides in HepG2 cells and the same three modified peptides identified in THLE2 cells by GMP Fury and MaxQuant. Whereas MSFragger was the fastest method, only one glycidamide-modified peptide in HepG2 cells and two in THLE2 cells were identified by this search engine (Table 1).

**Figure 3:**
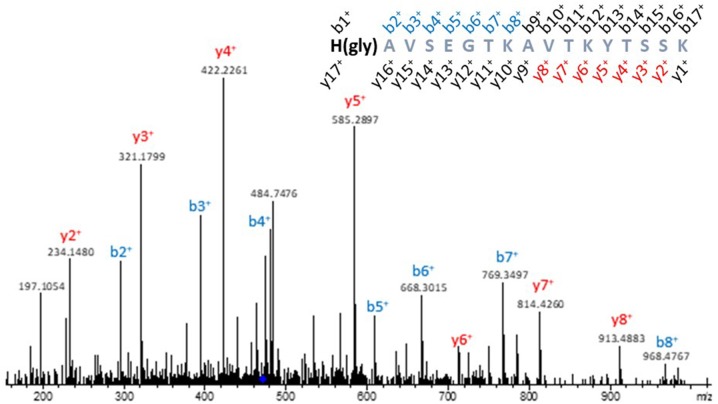
Representative MS/MS spectrum of a glycidamide-modified histone. MS/MS spectrum of the tetra charged ion at *m/z* 467.0030, corresponding to the ^110^H(glycidamide)AVSEGTKAVTKYTSAK^126^ peptide of Histone H2B. The 87.0320 Da mass increment, characteristic of glycidamide incorporation, could be observed in the b${}_{2}^{+}$ ion (*m/z* 296.1391) of the glycidamide-modified peptide, which confirmed H110 as the glycidamide binding site.

The position of glycidamide incorporation in each peptide was confirmed upon the MS/MS spectra of each ion (Figure 3, Supplementary Figure 2). Namely, in the MS/MS spectrum (Figure 3) of the tetra charged ion corresponding to the glycidamide-modified (*m/z* 471.0020) peptide ^110^HAVSEGTKAVTKYTSSK^126^ of Histone H2B, the 87.0320 Da mass increment, characteristic of glycidamide incorporation, is observed in the b^2+^ ion (*m/z* 296.1374). Taking into consideration that A is not a nucleophilic residue, the identification of this fragment ion confirmed H110 as the glycidamide binding site.

All glycidamide-modified peptides identified by the standard proteomics search engines were also identified by the metabolomics-inspired approach. Additionally, the tetra charged ion at *m/z* 580.3200, corresponding to the glycidamide-modified peptide ^74^IAGEASRLAHYNKRSTITSR^93^, was only identified by the metabolomics-inspired approach in THLE-2 cells (Table 1). The limited number of b and y ions present in the MS/MS spectrum of this glycidamide-modified peptide precluded the assignment of the exact residue of adduction. However, the identification of this peptide solely by our novel approach attests the advantage of the use of the metabolomics-inspired workflow over the standard proteomics approach.

**Figure 4:**
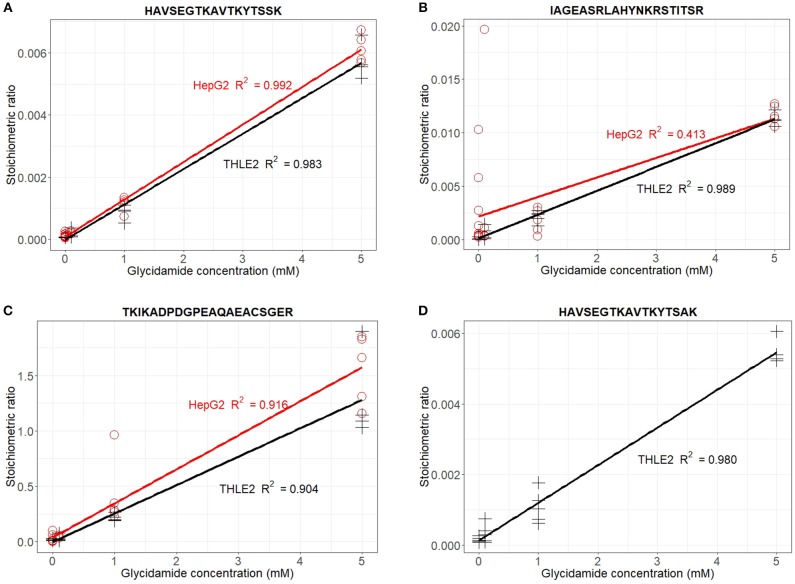
Scatter plots showing the relationship between glycidamide concentration and the stoichiometric ratios for each identified adduct in HepG2 (red) and THLE2 cells (black). **(A)** Stoichiometric ratios for **H**AVSEGTKAVTKYTSSK = ratio (triple charged *m/z* 627.667 + tetra charged *m/z* 417.002)/(triple charged *m/z* 598.6566 + tetra charged *m/z* 449.2441). **(B)** Stoichiometric ratios for IAGEASRLAHYNKRSTITSR = ratio *m/z* 580.3200/*m/z* 558.5612. **(C)** Stoichiometric ratios for TKIKADPDGPEAQAEA**C**SGER = ratio (triple charged *m/z* 754.0300 + tetra charged *m/z* 565.7740)/(tetra charged *m/z* 725.0163 + triple charged *m/z* 544.015). **(D)** Stoichiometric ratios for **H**AVSEGTKAVTKYTSAK = ratio *m/z* 467.0030 / *m/z* 445.2466.

Stoichiometric ratios were calculated for each peptide (Figure 4). With the exception of the peptide ^2^TKIKADPDGPEAQAEACSGER^22^ that presented a ratio of 15:10, all other modified-peptides presented ratios up to 10:1,000 at the maximum glyciamide dose. Importantly, there was a linear relationship between the stoichiometric ratio and the glycidamide concentration with *R*^2^ > 0.9 for all peptides except for the ^74^IAGEASRLAHYNKRSTITSR^93^ in HepG2 cells. These analyses confirmed the results obtained in the previous PLS models (Supplementary Figure 1) and proved the potential usefulness of the identified adducts as biomarkers of exposure to acrylamide if occurring *in vivo*.

There are multiple computational methods to identify adducts from MS/MS experiments (reviewed by Na and Paek, 2015). Conventional methods use database search engines in which experimental and theoretical MS/MS spectra are aligned after the introduction of a list with the known mass increments of the modifications (Mascot, X!Tandem) (Perkins et al., 1999; Craig and Beavis, 2004). Other advanced database-dependent strategies that are not restrictive to a predefined list of mass increments have been developed and include tolerant database search (e.g., MS Alignment, MSFragger) (Tsur et al., 2005; Kong et al., 2017), the use of *de novo* sequences (SIPDER, OpenSea) (Han et al., 2005; Searle et al., 2005) and the use of tag sequences (MODa MODi) (Kim et al., 2006; Na and Paek, 2015). However, as database-dependent methods, all those approaches rely on the availability and quality of MS/MS spectra and on the sequence databases. Moreover, database-dependent methods tend to be time-consuming and report a high rate of false positives. Too much time is put into the identification of adducted and non-adducted peptides with no prior knowledge of their statistical or biological relevance.

Our approach can be classified as a database-independent method since individual spectra are not assigned to peptide sequences. As in other spectral-pair approaches methods (e.g., ModifiComb, DeltAMT, Peptoscope, P-Mod) (Hansen et al., 2005; Savitski et al., 2006; Potthast et al., 2007; Fu et al., 2011) we started by assuming that the modified and unmodified versions of a peptide are present simultaneously in the sample and use the mass increment to find the modified ion. However, with few exceptions (e.g., DeltAMT), most of spectral-pair approaches work with MS/MS data, meaning that only those ions that undergo MS/MS will be considered for the analysis.

One of the advantages of our approach is the identification of potential covalent adduct ions strictly working with full scan MS data using MZmine, which is a user-friendly open-source software for mass-spectrometry data processing. We should, however, state that by using the adduct search tool of MZmine, we are only going to be able to identify the adducted peptides whose unmodified peptide is also present in the ion list afforded upon MS analysis. This means that if a missed-cleavage occurs due to the presence of the modification, most probably this modified peptide is not going to be identified by the methodology followed. However, taking into consideration that a list of *m/z* values of tryptic peptides with two or more miss-cleavages is something very easy to obtain, the calculation of the *m/z* values expected for the corresponding adducted peptides can be easily obtained and used to match with the list of experimentally obtained *m/z* values. Actually, the combined use of the approach herein presented with theoretical calculations, can result in an increased number of potentially modified peptides.

Regarding the future application of our workflow for the identification of covalent adducts formed in humans, the statistical analysis is expected to be more challenging since exposure is not controlled and the presence of confounding factors (e.g., age, gender, metabolizing enzymes polymorphisms) is anticipated. Nonetheless, a good study design can overcome these difficulties. In contrast, the low levels of covalent adducts *in vivo* is *per se* the major difficulty in adductomics studies, which cannot be solved by the current database-dependent methods. Therefore, our approach can constitute a prospective solution for the identification of covalent adducts in humans.

Taken together these results, our metabolomics-inspired workflow has several advantages when compared with database-dependent and -independent methods reported to date: (1) works with full scan MS data and not MS/MS data, so it is more inclusive because it does not depend on the availability or quality of MS/MS data; (2) the identification of potential adducted peptides is performed with MZmine that is a user-friendly open-source software; and (3) statistical analysis is used to select the potential adducts of interest for adductomics studies, which can increase the accuracy of the findings and thus reduce the false discovery rate. Actually, the most distinctive feature of our methodology over the methods previously developed for covalent adducts identification is the use of statistics prior to adducts identification. While database-dependent or independent methods are focused on improving peptide identifications without considering their statistical or biological relevance, our method firstly selects the potential adducts that are relevant for the study endpoints applying statistics and only after that goes for the identification of those adducts. An additional and imperative advantage of the statistical analysis is that it can identify all potential factors (e.g., cell lines, doses) that are influencing the adducted peptides. This is crucial in any adductomics study.

## Conclusion

**Figure 5:**
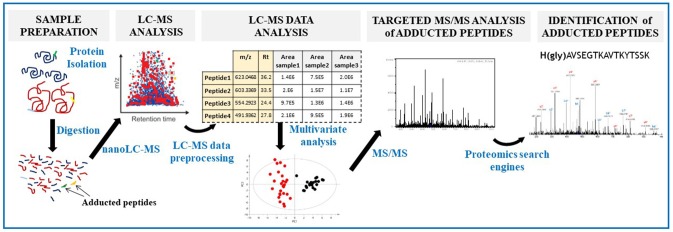
Workflow of our metabolomics-based approach to identify protein covalent modifications. After protein digestion, we propose a workflow of three steps: (1) sample acquisition in full scan mode (MS1 level); (2) LC-MS data preprocessing (to yield a matrix with all detected ions with their corresponding *m/z*, retention time and peak area) followed by statistical analysis; (3) targeted MS/MS analysis of those statistically selected ions.

We present a new metabolomics-inspired data processing approach for the identification of covalently-modified peptides that is fast, sensitive and allows to perform any statistical analysis. Thus, this method enables the identification of low abundant adducted peptides and all factors influencing the formation and levels of covalent adducts. We herein propose a new adductomics workflow consisting on 3 steps (Figure 5): (1) data acquisition in full scan mode to maximize the sensitivity; (2) LC-MS data preprocessing followed by statistical analysis to reveal those ions (adducts) that differentiate negative samples from positive samples (non-exposed vs. exposed or healthy vs. disease); (3) targeted MS/MS acquisition of the statistically significant ions for adduct identification. This approach is expected to result in higher quality MS/MS spectra of low level adducted-peptides, when compared with DDA and DIA approaches, thereby enhancing the chances of identifying low abundant adducted peptides in biological samples. This will exponentially increase the number and accuracy of findings for all fields of adductomics application, encompassing epigenetic and toxicological studies.

## Data Availability

The mass spectrometry dataset analyzed for this study can be found in the ProteomeXchange Consortium via the PRIDE partner repository with the dataset identifier PXD013683 and 10.6019/PXD013683.

## Author Contributions

AA planned the work. JS and JM supervised the cell assays and data processing, respectively. JN, CC, CN, and SG-F performed the experiments. JN and JM processed the raw data. JN, JM, and AA wrote the article. JM and AA critically revised the manuscript. All authors approved the final version of the manuscript.

### Conflict of Interest Statement

The authors declare that the research was conducted in the absence of any commercial or financial relationships that could be construed as a potential conflict of interest.
