# Supplementary material for: A Metabolomics-Inspired Strategy for the Identification of Protein Covalent Modifications
Source: Front Chem. 2019 Jul 31;7:532. doi: 10.3389/fchem.2019.00532 (PMC6684772; doi:10.3389/fchem.2019.00532)
Supplement: Supplementary file 1 [file Data_Sheet_1.docx]

Supplementary Material

# Supplementary Figures and Tables

## Supplementary Figures

**A**

**B**

**Supplementary Figure 1.** Observed *versus* predicted plots of the partial least square (PLS) analysis with glycidamide concentration as the dependent variable. **(A)** PLS model for HepG2 cells (p= 3.73e-008, R^2^X = 45%, R^2^Y = 93%). **(B)** PLS model for THLE2 cells, (p= 4.59e-008, R^2^X = 21%, R^2^Y = 93%).

**A
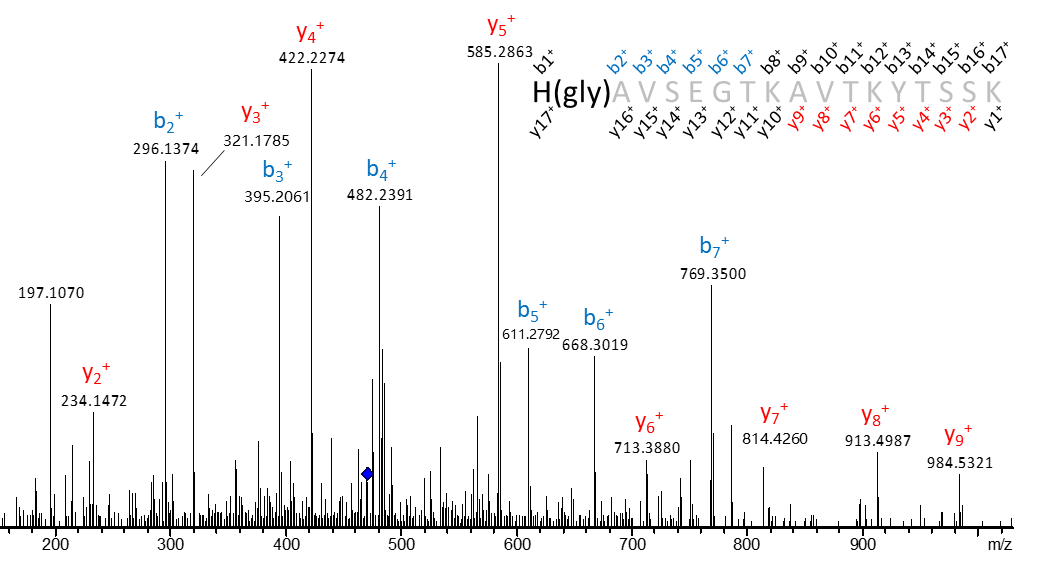
**

**
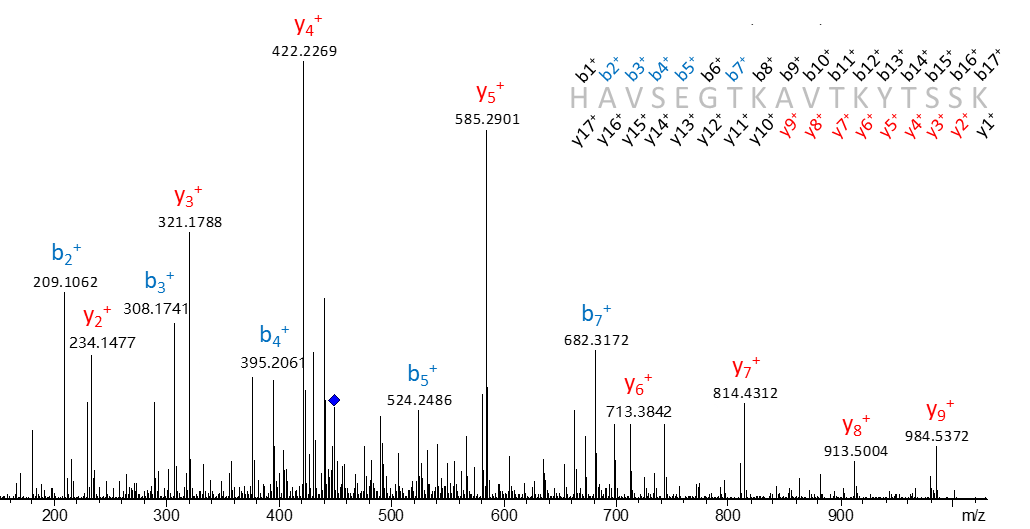
**

**B**
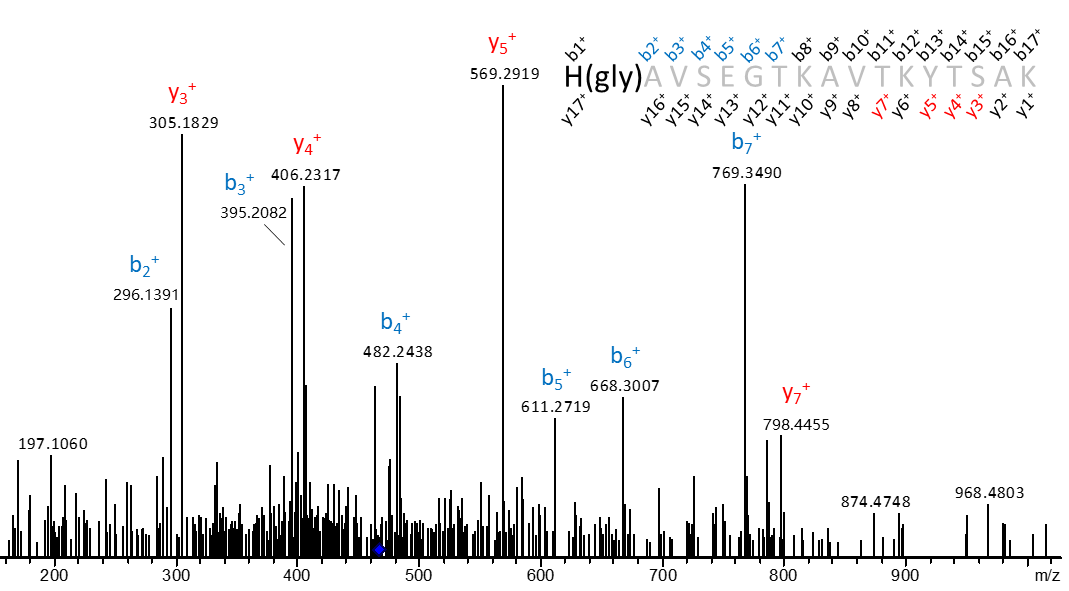


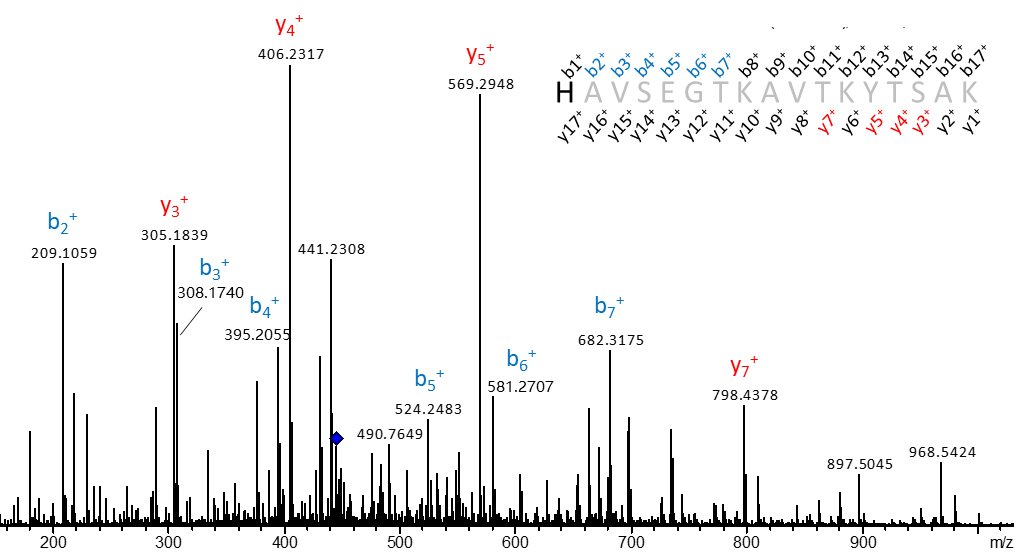


**
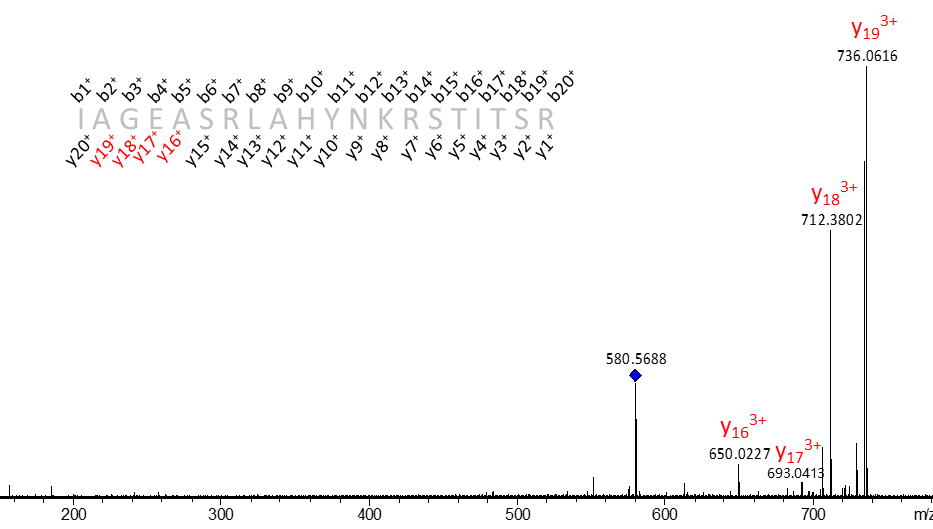
C**

**
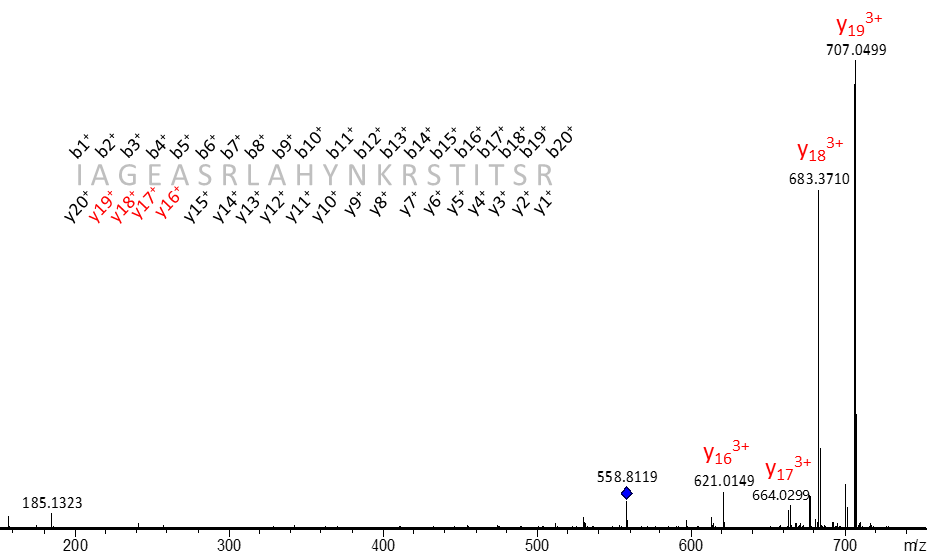
**

**
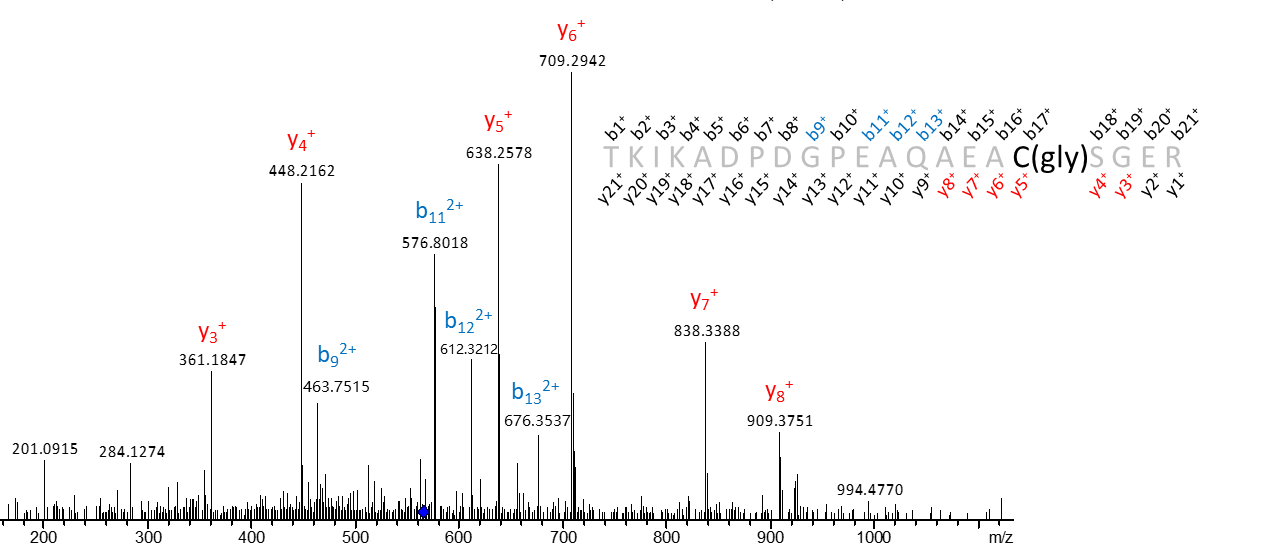
D
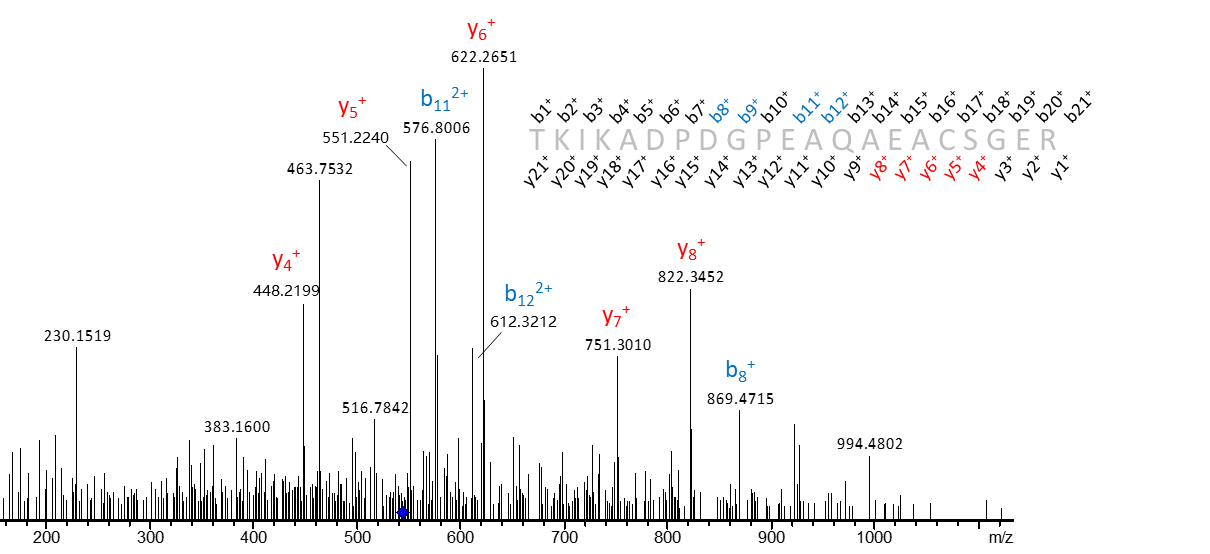
**

**Supplementary Figure 2. MS/MS spectra of the identified glycidamide-modified peptides and their corresponding non-modified peptides. (A)** MS/MS spectra of the tetra charged ions corresponding to the modified (*m/z* 471.0020) and non-modified (*m/z* 449.2441) peptides of ^110^HAVSEGTKAVTKYTSSK^126^ of Histone H2B. The 87.0320 Da mass increment, characteristic of glycidamide incorporation, could be observed in the b_2_^+^ ion (*m/z* 296.1374) of the glycidamide-modified peptide, which confirmed H110 as the glycidamide binding site. **(B)** MS/MS spectra of the tetra charged ions corresponding to the modified (*m/z* 467.0030) and non-modified (*m/z* 445.2453) peptides of ^110^HAVSEGTKAVTKYTSAK^126^ of Histone H2B. The 87.0320 Da mass increment, characteristic of glycidamide incorporation, could be observed in the b_2_^+^ ion (*m/z* 296.1391) of the glycidamide-modified peptide, which confirmed H110 as the glycidamide binding site. **(C)** MS/MS spectrum of the tetra charged ion corresponding to the modified (*m/z* 580.3200) and non-modified (*m/z* 558.5597), corresponding to the ^74^IAGEASRLAHYNKRSTITSR^93^ peptide of the Histone H2B.  **(D)** MS/MS spectra of the tetra charged ions corresponding to the modified (*m/z* 565.7724) and non-modified (*m/z* 544.0102) peptides of ^2^TKIKADPDGPEAQAEACSGER^22^ of the H/ACA ribonucleoprotein complex subunit 2. The 87.0320 Da mass increment, characteristic of glycidamide incorporation, could be observed in the y_5_^+^ ion (*m/z* 638.2578) of the glycidamide-modified peptide, which confirmed C18 as the glycidamide binding site.

## Supplementary Tables

**Supplementary Table 1. Accession numbers of all histone variants corresponding to the identified peptides.**

| **Peptide** | **Protein** | **Uniprot Entry** |
| --- | --- | --- |
| HAVSEGTKAVTKYTSSK | Histone H2B type 1-C/E/F/G/I | P62807 |
|  | Histone H2B type 1-M | Q99879 |
|  | Histone H2B type 2-F | Q5QNW6 |
|  | Histone H2B type 1-H | Q93079 |
|  | Histone H2B type 1-N | Q99877 |
|  | Histone H2B type 1-D | P58876 |
|  | Histone H2B type 1-O | P23527 |
|  | Histone H2B type 2-E | Q16778 |
|  | Histone H2B type 1-B | P33778 |
|  | Histone H2B type 1-L | Q99880 |
|  | Histone H2B type 3-B | Q8N257 |
|  | Histone H2B type 1-A | Q96A08 |
| HAVSEGTKAVTKYTSAK | Histone H2B type 1-K | O60814 |
|  | Histone H2B type 1-J | P06899 |
|  | Histone H2B type F-S | P57053 |
| IAGEASRLAHYNKRSTITSR | Histone H2B type 1-C/E/F/G/I | P62807 |
|  | Histone H2B type 1-M | Q99879 |
|  | Histone H2B type 2-F | Q5QNW6 |
|  | Histone H2B type 1-H | Q93079 |
|  | Histone H2B type 1-N | Q99877 |
|  | Histone H2B type 1-D | P58876 |
|  | Histone H2B type 1-O | P23527 |
|  | Histone H2B type 2-E | Q16778 |
|  | Histone H2B type 1-B | P33778 |
|  | Histone H2B type 1-K | O60814 |
|  | Histone H2B type 1-J | P06899 |
| TKIKADPDGPEAQAEACSGER | H/ACA ribonucleoprotein complex subunit 2 | Q9NX24 |
